# Involvement in Specific HIV Risk Practices among Men Who Use the Internet to Find Male Partners for Unprotected Sex

**DOI:** 10.1155/2013/826039

**Published:** 2013-03-25

**Authors:** Hugh Klein

**Affiliations:** Kensington Research Institute, 401 Schuyler Road, Silver Spring, MD 20910, USA

## Abstract

*Purpose*. Men who have sex with other men (MSM) account for more than one-half of all new HIV infections in the USA. This study reports on the prevalence of a variety of HIV risk behaviors in one specific subpopulation of risk-seeking MSM. *Methods*. The study was based on a national sample of 332 MSM who use the Internet to find partners for unprotected sex. Data collection was conducted via telephone interviews between January 2008 and May 2009. *Results*. Unprotected oral and anal sex was commonplace among study participants. Men engaged in a large number of other risky behaviors as well, including having had multiple recent sex partners (mean number = 11), simultaneous double-penile penetration of the anus (16%), eating semen out of another man's anus (17%), engaging in multiple-partner sexual encounters (47%), engaging in anonymous sex (51%), and having sex while “under the influence” (52%). *Conclusions*. HIV intervention and prevention programs need to address numerous behaviors that place MSM at risk for contracting/transmitting HIV. Merely focusing on unprotected anal sex does a disservice to members of this community, who typically engage in many types of behavioral risks, each of which requires addressing if HIV transmission rates are to be reduced.

## 1. Introduction

In recent years, evidence has been mounting to suggest that men who have sex with other men (MSM) increasingly are turning to the Internet to meet partners for sex. For example, in a sample of gay men who were recruited into a health promotion study via gay-oriented Internet websites, Bolding and colleagues' [1] multivariate analysis revealed that the amount of risky sex in which men engaged was a significant predictor of their use of Internet websites to locate sex partners. Bolding et al. also reported that 47% of the men in their sample said that, when they wanted to identify potential sex partners, they preferred using websites to frequenting bars or other “offline” venues. In another study [2], among men actively using the Internet as a means of locating potential sex partners, 97% reported actually having met someone online for sex, and 86% said that they used Internet MSM sex sites at least once a week to identify possible partners. Ogilvie and colleagues [3] found that MSM who used the Internet to initiate sexual relationships reported having had more sex partners during the previous year than their counterparts who did not use the Internet for this purpose. Berg [4] noted that, compared with men who did not engage in bareback sex, barebacking men said that they met their sex partners online rather than offline more than twice as frequently. More recently, based on their study of gay and bisexual men traveling to a major Gay Pride celebration, Benotsch and colleagues [5] found that men who used the Internet to set up “dates” prior to their travel acknowledged having more sex partners and were more likely to report having sex with a new partner than men who did not use the Internet to identify potential sex partners prior to their vacation travel.

Not only are MSM using the Internet more frequently to meet potential sex partners, but they also appear to be using it specifically to find partners with whom they can engage in risky sexual acts. For example, based on their research on MSM in the Seattle area, Menza and colleagues [6] reported that the proportion of anal sex partners who had met online increased significantly from 2003 to 2006. Halkitis et al. [7] cited Internet websites and chat rooms as key sources that are partly responsible for the upsurge of unprotected sexual activities that they have observed among gay and bisexual men in the New York City area. In their study of rural versus urban MSM, Kakietek et al. [8] found that Internet use was associated with an increased risk for engaging in unprotected anal sex overall and, in particularly, an increased risk for engaging in unprotected insertive anal sex for the rural men in their sample. Rosser and colleagues's [9] study of men visiting one of the Internet's most-popular gay-oriented websites noted that reliance upon the Internet to identify sex partners was associated with approximately a doubling of the number of times engaging in unprotected anal intercourse with male partners.

It should be noted, however, that not all studies have found that using the Internet to meet sex partners is associated with increased risk. Examples include Chiasson et al. [10], Coleman et al. [11], Mustanski [12], and Mustanski and Newcomb [13]. Moreover, mixed results have been reported by other researchers (e.g., [14]), whose work found that barebacking MSM spent more time on the Internet looking for sex while simultaneously engaging in greater serosorting practices. Mixed findings were also reported by Jenness and colleagues [15], whose work showed that it was not meeting partners online per se that was associated with elevated HIV risk involvement among MSM, but rather, it is the combination of meeting some partners online and others offline that led to heightened risk for HIV. Although their findings were of borderline statistical significance, Berry et al. [16] concluded that using the Internet to identify sex partners is associated with an increased risk of engaging in unprotected anal intercourse among HIV-negative MSM, but not among HIV-positive MSM.

One argument that is made occasionally is that men using the Internet for the purported purpose of identifying sex partners may not, in actuality, be using it for that purpose. Instead, it has been suggested that the act of searching through profiles online may be an erotic act for some men, who have no realistic intention of meeting in person anyone with whom they have interacted online. For these persons, posting a sex-related profile online or responding to other people's online postings may be expressions of fantasy or manifestations of symbolic preferences rather than actual sexual desires. Published studies have shown that fantasizing about engaging in unprotected sex is related to lower intentions to use condoms during actual sexual situations [17] and, thus, is not a purely harmless behavior in and of itself. Moreover, research has also shown that MSM who use the Internet with the intention/hope of meeting partners face to face rather than limiting themselves exclusively to the online interaction are more likely to discuss unprotected sex and less likely to talk about sexual safety with those online-met partners [18]. Furthermore, based on a large-scale content analysis study of one of the largest bareback-focused websites on the Internet, Klein [19] concluded that “men who use the Internet to locate sexual partners are very likely to meet up with such individuals for sex (i.e., their ads/profiles are, far more often than not, not posted purely for fun, but rather with sexual hook-ups in mind) suggest[ing] that there may not be a great disconnect between ad/profile content and behavioral practices.”

This leaves open the question of what risky behaviors are practiced “in real life” (as distinguished from fantasized sexual behaviors that are promised in online profiles or “cybersex” behaviors that are discussed online in chats/conversations that do not lead to in-person sexual encounters) by MSM who use the Internet to identify partners for sex. Although numerous authors (cited previously) have reported that identifying sex partners via the Internet is associated with involvement in risky behaviors vis-a-vis HIV, their research has provided very little information about the extent to which men seeking sex online engage in specific behaviors that place them or their sex partners at risk for contracting HIV. This is the subject of the present report. 

This paper examines the extent to which one specific subpopulation of MSM (namely, those who use the Internet for the express purpose of finding partners with whom they can engage in unprotected sex) engages in various behaviors that place them and their sex partners at risk for contracting HIV. The research contributes to the scholarly literature by documenting the prevalence of specific risk practices undertaken when members of this population use the Internet to find sex partners. Moreover, this paper provides information about a variety of risk behaviors that have not been discussed much in the scholarly literature, but which are practiced with relative frequency by MSM. Included among these less-well-researched/discussed high-risk practices are ejaculating internally during sex, simultaneous double-penile penetration, oral-anal contact, sharing of previously used but uncleaned sex toys, multiple-partner sexual encounters, the number of drug-related problems experienced, and men's preferences for engaging in sexual relations while under the influence of alcohol and other drugs. Additionally, throughout the paper, comparisons of risk involvement are provided for men who are HIV-positive and those who are HIV-negative, as HIV serostatus has been found to be a strong predictor of risk behavior practices among MSM [20, 21]. The paper concludes by discussing the implications of these findings for prevention and intervention.

## 2. Methods

### 2.1. Procedures

The data reported in this paper come from *The Bareback Project*, a National Institute on Drug Abuse-funded study of men who use the Internet specifically to find other men with whom they can engage in unprotected sex. The data were collected between January 2008 and May 2009. A total of 332 men were recruited from 16 different websites. Some of the sites catered exclusively to unprotected sex (e.g., http://www.bareback.com/, http://www.barebackrt.com/) and some of them did not but made it possible for site users to identify which persons were looking for unprotected sex (e.g., http://www.men4sexnow.com/). In order to be eligible to participate in the study, men had to be aged 18 or older—a requirement that eliminated almost nobody because all of the sites used for this study required men to certify their age as that of an adult before they could become members or site users. In addition, participants had to be residents of the United States, so as to keep this a USA-focused study.

A nationwide sample of men was derived, with random selection being based on a combination of the first letter of the person's online username, his race/ethnicity (as listed in his profile), and the day of recruitment. Each day, members of the research staff working on recruitment had three letters or numerals assigned to them for their use that day. These letters and numerals were assigned randomly, using the software available at http://www.random.org/ (substituting the numbers 1 to 26 to represent, sequentially, the letters of the alphabet, and then using numbers after that to represent numerals). The first letter/numeral was restricted for use for recruiting Caucasian men only; the last two letters/numerals were to be used exclusively for recruiting men of color. (This oversampling technique for racial minority group men was adopted so as to compensate for the fact that men of color, especially African-American men, are more difficult to recruit into research studies than their Caucasian counterparts are.) In order for a particular person to be approached and asked to participate in the study, these letters/numerals had to correspond to the first letter/numeral of that individual's profile and that person's race/ethnicity, as stated in his profile, had to be a match for the Caucasian-versus-racial-minority-group-member designation on the daily randomization listing. 

On recruitment sites where it was possible to know who was online at the time the recruiter was working, selection of potential study participants came from the pool of men who happened to be logged onto the site during the time block when the recruiter was working. All men who were online at the time the recruiter was working and whose profile name began with the appropriate letter/numeral were eligible to be approached. On recruitment sites where it was not possible to know who was online at the time the recruiter was working, ZIP codes were used to narrow down the pool of men who could be approached. To do this, in addition to the daily three letters/numerals that were assigned randomly to each recruiter throughout the study, each day, ten five-digit numbers were also assigned to each recruiter (five to be used for Caucasian men, five to be used for men of color). These five-digit numbers were random number combinations generated by the http://www.random.org/ software, and they were used in this study as proxies for ZIP codes. Recruiters entered the first five-digit number into the website's ZIP code search field (which site users typically utilized to identify potential sex partners who resided within a specified radius from their residence), selected a five-mile radius, and then viewed the profile names of all men meeting those criteria who had logged onto that site within the previous 24 hours. Those men were eligible to be invited to participate, and their profiles were reviewed for the letter/numeral match described previously for men who were online at the time that recruiters were working.

Recruitment efforts were undertaken seven days a week, during all hours of the day and nighttime, variable from week to week throughout the duration of the project. This was done to maximize the representativeness of the final research sample, in recognition of the fact that different people use the Internet at different times. 

Depending upon the website involved, men were approached initially either via instant message or email (much more commonly via email). A brief overview of the study was provided as part of the initial approach and informed consent-related procedures, and all men were given the opportunity to ask questions about the study before deciding whether or not to participate. ^1^ A website link to the project's online home page was also made available, to provide men with additional information about the project and to help them feel secure in the legitimacy of the research endeavor. Interviews were conducted during all hours of the day and nighttime, seven days a week, based on interviewer availability and participants' preferences, to maximize convenience to the participants. Over the course of the 17-month study period, no person could be approached more than five times (once every 90 days) and asked to participate. Anyone who specifically indicated that he was not interested in taking part in the study was listed on the daily-updated contacts list as “no further contact—refused” and was not contacted again.

Participation in the study entailed the completion of a one-time, confidential telephone interview covering a wide array of topics. The decision to conduct the data collection via telephone interviews rather than via anonymous online surveys was made for a number of reasons. First, telephone interviews allowed the research team members to establish rapport with respondents, and this was deemed critical in light of the length of the questionnaire and the very personal nature of the questions being asked. Second, using telephone interviews enabled the research team to make sure that study participants understood all of the questions (something that cannot be achieved when online survey techniques are used) and helped people to “think through” some of the more complex questions asked during the interview. Third, *The Bareback Project *was a mixed-methods study, involving the collection of both quantitative and qualitative data. The latter would have been precluded had only an online survey been implemented.

The questionnaire was developed specifically for use in *The Bareback Project*, with many parts of the interview derived from standardized scales previously used and validated by other researchers. The interview covered such subjects as degree of “outness,” perceived discrimination based on sexual orientation, general health practices, HIV testing history and serostatus, sexual practices (protected and unprotected) with partners met online and offline, risk-related preferences, risk-related hypotheticals, substance use, drug-related problems, Internet usage, psychological and psychosocial functioning, childhood maltreatment experiences, HIV/AIDS knowledge, and some basic demographic information. Interviews lasted an average of 69 minutes (median = 63, s.d. = 20.1, range = 30–210). Men who completed the interview were compensated $35 for their time. Prior to implementation in the field, the research protocol was approved by the institutional review boards at Morgan State University, where the principal investigator and one of the research assistants were affiliated, and George Mason University, where the other research assistant was located.

### 2.2. Measures Used

Men were asked separate questions about the number of different men with whom they had had *any *kind of sex during the past 30 days (continuous measure) and over the course of their lifetimes (continuous). Men who initially responded “don't know” were instructed “Please take a moment and think about it, and give me a number that you know is definitely safe—that is, the number of men with whom you have had any kind of sex that you know “It is definitely no less than *|x|* persons. For example, you definitely had sex with no fewer than 3 men, or no fewer than 30 men, or no fewer than 300 men, and so forth.” In this manner, self-reports of the number of sex partners range from accurate to conservative estimates.

For insertive oral sex, receptive oral sex, insertive anal sex, and receptive anal sex, separate questions were asked about the number of times engaging in each behavior during the previous 30 days (continuous), the number of partners with whom men had engaged in each behavior during that time frame (continuous), the number of those times in which the insertive partner wore a condom (continuous), and the number of those times that the receptive partner received ejaculatory fluid directly inside of his mouth or anus (continuous). For these specific behaviors, men were also asked how many of the men in question were people who had met online, how many times they had each type of sex with those online-met partners, how many of those situations involved the use of a condom, and how many of those sex acts involved internal ejaculation.

Subsequently, participants were asked several questions (all using a past-30-days time frame of reference, all continuous measures) about their involvement in other sexual practices. The first questions asked about “the number of times that you and another man both put your penises into another man's anus at the same time” (i.e., double fucking), “the number of times that two men both put their penises into your anus at the same time” (i.e., being double fucked), and for both of these behaviors, the number of times that at least one of the insertive partners ejaculated directly inside of the receptive partner's anus. Men were also asked “How many times did you have sex where you put your mouth or tongue onto or inside of another man's anus?” (i.e., rimming) and then “How many of these times had you or someone else ejaculated into this man's anus beforehand?” (i.e., felching) and then “And how many of those times did you then feed the semen to one of your sex partners by a kiss or drooling it onto or into his mouth?” (i.e., snowballing). Information about sharing sex toys was obtained by asking men “How many times have you had sex where a sex toy like a dildo or a butt plug was used inside of you?” and then “How many of those times had that item been used by your partner beforehand, without being cleaned before you used it?” and then “How many times of those times did you give the sex toy to your partner to use, without cleaning it first?” Information about “pimping out” a sex partner or “being pimped out” by a sex partner was gathered by asking “How many times have you had sex where your partner brought several other men to have sex with you? Some people call this being passed around or being pimped out by your partner” and then “How many times have you had sex where you brought several men to have sex with your partner? Some people call this passing around the partner or pimping him out.” Men's practices of multiple-partner sex were ascertained by asking “How many times have you had any kind of sex in a three-way arrangement, where you and your partner had one other man join you for sex?” and then “How many of those times were condom used [by anyone involved]?” and then “How many times have you had any kind of sex involving more than a total of three people—what some people call group sex or orgies?” and then “How many of those times were condoms used [by anyone involved]?” To learn about men's anonymous sex practices, the questions read as follows: “Some people like to have anonymous sex—that is, sex with persons they know nothing about—and some people do not like to do this. Do you like having anonymous sex?” and then “How many times during the past 30 days have you had anonymous sex of any kind with someone?”.

Drug use behaviors were inquired about in a separate section of the questionnaire. There, men were asked about lifetime usage (yes/no), age of first use (continuous), number of days of use during the preceding 30 days (continuous), number of times using on a “typical” day of use during the past 30 days (continuous), and the number of times using shortly before having sex with someone or while having sex with someone (continuous). These questions were asked separately for alcohol, marijuana, powdered cocaine, crack cocaine, heroin or other opiates, hallucinogens, ecstasy, club drugs other than ecstasy (e.g., ketamine/“Special K,” GHB, etc.), methamphetamine, Viagra or its equivalent (excluded from the present paper's analysis of “illegal drug use”), and sedatives or depressant drugs that were not prescribed by a physician. The format for these questions was derived from the substance use section of the widely used, validated, and reliability-tested *Risk Behavior Assessment* [22]. Additionally, men were asked “Suppose for the moment that you could have access to any type of drugs you wanted—alcohol, cocaine, whatever. Which types of drugs, if any, would you yourself most like to use shortly before you had sex with someone or while you were having sex?” This question was constructed specifically for use in this study. Men were also asked about their lifetime and past-30-days experiences with 14 types of substance abuse problems (each asked in yes/no format), including needing more of a drug in order to get the same effect previously experienced, having problems with family members as a result of one's own substance use/abuse, being unsuccessful in one's efforts to quit or curtail one's own drug intake, and experiencing withdrawal symptoms when unable to get alcohol or another drug to use. These measures were derived from the *DSM-IV-TR* [23] for the diagnosis of substance abuse and substance dependency, but not all of the diagnostic criteria were included, nor were details about the clustering of substance abuse-related symptoms within the *DSM-IV-TR*'s specified 12-month period.

### 2.3. Statistical Analysis

Much of this work is based on the presentation of descriptive statistics, with the appropriate confidence intervals (CI_95_) and/or standard deviations provided to accompany the percentages and means reported. Parts of the analysis, especially those whose results are summarized in Tables 3 and 5, entailed comparing men who were HIV-negative and those who were HIV-positive on the prevalence and/or extent of involvement in various risky practices. Whenever the dependent variable was continuous in nature (e.g., number of sex partners, number of times engaging in a particular type of sex), Student's *t*-tests were used. Whenever the dependent variable was dichotomous or categorical in nature (e.g., recent involvement in a particular type of risky sex, recent use of a particular illegal drug), chi-square tests were performed. Throughout Section 3, findings are reported as statistically significant whenever *P* < .05.

## 3. Results

### 3.1. Sample Characteristics

In total, 332 men participated in the study. They ranged in age from 18 to 72 (mean = 43.7, s.d. = 11.2, median = 43.2) (see Table 1). Racially, the sample is a fairly close approximation of the American population, with 74.1% being Caucasian, 9.0% each being African American and Latino, 5.1% self-identifying as biracial or multiracial, 2.4% being Asian, and 0.3% being Native American. The large majority of the men (89.5%) considered themselves to be gay and almost all of the rest (10.2%) said they were bisexual. On balance, men participating in *The Bareback Project *were fairly well educated. About 1 man in 7 (14.5%) had completed no more than high school, 34.3% had some college experience without earning a college degree, 28.9% had a bachelor's degree, and 22.3% were educated beyond the bachelor's level. Slightly more than one-half of the men (59.0%) reported being HIV-positive; most of the rest (38.6%) were HIV-negative.

Table 1 also shows that men in this study used the Internet to find sex partners with great regularity. More than two-thirds of the men (68.3%) reported looking online specifically for bareback sex partners at least three days a week, on average, and even more of them (84.0%) said that they used the Internet three or more days each week to find sex partners on sites that were not bareback focused. As a general rule, the more frequently men reported looking online for sex partners, the more time they tended to spend on each of the days that they were online engaged in this pursuit.

### 3.2. Sexual Activity

The large majority of the men (88.9%, CI_95_ = 86.5–92.2%) participating in *The Bareback Project *had been sexually active during the month prior to interview. Sexually active men reported an average of more than 11 sex partners (mean = 11.3, s.d. = 16.7, range = 1–151) during the past month. Over the course of their lifetime, the bottom quartile of men in the study reported having had anywhere from 1 to 79 sex partners, the next-higher quartile of men reported 80 to 275 sex partners, the next-higher quartile of men reported 276 to 975 sex partners, and the top quartile reported anywhere from 976 to 20,000 sex partners. ^2^

Men who were HIV-negative and those who were HIV-positive were equally likely to report having been sexually active during the month prior to interview (88.2% versus 89.3%, *χ* ^2^_1df_ = 0.09, n.s.). Sexually active HIV-positive men reported significantly more sex partners during the month prior to interview than their HIV-negative counterparts (13.1 versus 8.7, *t* = 2.25, *P* = .025). HIV-positive men also reported more than three times as many lifetime sex partners than HIV-negative men did, on average (1648.5 versus 506.1, *t* = 4.20, *P* < .001).

Table 2 presents information pertaining to various sexual behaviors among men in the sample. Almost all of the sexually active men (94.2%, CI_95_ = 91.6–96.9%) reported having performed oral sex on another man during the month prior to interview. On average, they reported having done this with 7.6 different partners (s.d. = 13.1) a total of 12.2 times (i.e., usually once or twice with each man; s.d. = 17.6). Most of the time (54.7%, CI_95_ = 48.8–60.5%), the man accepted semen orally during these oral sex behaviors. Approximately one-half (55.4%) of men's partners for this type of sex were people they had met online.

Table 3 presents information comparing sexual risk behavior involvement of HIV-negative and HIV-positive men. This table shows that HIV-negative and HIV-positive men alike were highly likely to report having performed oral sex on another man during the month prior to interview (92.5% versus 95.4%, *χ* ^2^_1df_ = 1.12, n.s.). HIV-positive men engaged in this behavior with significantly more partners than their HIV-negative counterparts did (8.9 versus 5.7, *t* = 2.07, *P* = .040). Consistent with this, HIV-positive men performed oral sex on their partners significantly more times during the month prior to interview than their HIV-negative counterparts did (14.5 versus 8.8, *t* = 2.70, *P* = .007), with both groups engaging in this behavior an average of once or twice with each partner. As Table 3 shows, HIV-positive and HIV-negative men were about equally likely to have performed oral sex on men they had met online (80.8% versus 80.2%, *χ* ^2^_1df_ = 0.02, n.s.). Both groups were, from a statistical standpoint, equally likely to have performed oral sex on another man without the use of a condom (94.9% versus 90.8%, *χ* ^2^_1df_ = 1.82, n.s.). HIV-positive men, however, were significantly more likely than HIV-negative men to accept their partner's semen in their mouths when performing oral sex (59.9% versus 46.9%, *χ* ^2^_1df_ = 4.57, *P* = .033). In the interest of conserving space and reducing verbiage, throughout the remainder of Section 3, findings for HIV-negative and HIV-positive respondents will be highlighted only when statistically significant differences were observed.

As Table 2 shows, the large majority (90.5%, CI_95_ = 87.2–93.8%) of sexually active study participants said that another man had performed oral sex on them during the 30 days prior to interview. On average, they said that 6.4 men had done this (s.d. = 9.1) a total of 10.9 times (again, typically averaging once or twice per sex partner; s.d. = 14.0). Most of the time (55.3%, CI_95_ = 49.3–61.2%), men said that they did not ejaculate into their partner's mouth during oral sex. Approximately one-half (54.9%) of men's partners for this type of sex were people they had met online.

Most of the sexually active men participating in *The Bareback Project *(64.1%, CI_95_ = 58.6–69.5%) said that they had performed insertive anal sex on another man during the month prior to interview. They reported having done this with approximately four men on average (mean = 4.2, s.d. = 7.3), approximately two or three times per partner (mean = 10.5, s.d. = 12.7). Most of the time (69.3%, CI_95_ = 62.7–75.8%), men said that they ejaculated directly into their partner's anus during these activities. Men said that most (62.4%) of the people with whom they recently performed insertive anal sex were people they had met via the Internet. As Table 3 illustrates, although HIV-positive and HIV-negative men were comparably involved in most aspects of insertive anal sex, the data did reveal that HIV-positive men were more likely than HIV-negative men to say that they had engaged in unprotected insertive anal sex during the preceding month (64.0% versus 50.8%, *χ* ^2^_1df_ = 5.09, *P* = .024).

A comparable percentage of the sexually active men (67.5%, CI_95_ = 62.1–72.8%) indicated that they had engaged in receptive anal sex during the preceding 30 days (see Table 2). On average, they had done this with four different sex partners (mean = 3.9, s.d. = 6.3) approximately two or three times each (mean = 9.3, s.d. = 11.4). The majority of the time (74.4%, CI_95_ = 68.3–80.4%), men said that their partners ejaculated directly inside of their anus. Most (58.6%) of the people with whom they reported recently having engaged in receptive anal sex were partners they had met online.

Table 3 shows that, when it came to receptive anal sex, HIV-negative and HIV-positive men often differed from one another. HIV-negative men were less likely to report this practice during the previous month (60.8% versus 72.0%, *χ* ^2^_1df_ = 4.04, *P* = .044). When they did engage in this behavior, HIV-negative men were less likely to engage in it without the use of condoms (51.7% versus 69.1%, *χ* ^2^_1df_ = 9.23, *P* = .002) and they were less likely to allow one of their partners to ejaculate directly into their anus (65.8% versus 79.4%, *χ* ^2^_1df_ = 4.49, *P* = .034).

### 3.3. Risky Sexual Practices

Two-thirds of the sexually active men (66.7%, CI_95_ = 61.3–72.1%) reported no condom whatsoever during the preceding month. HIV-positive men were significantly more likely than HIV-negative men to report no condom use whatsoever during the previous month (71.3% versus 60.0%, *χ* ^2^_1df_ = 4.06, *P* = .044). The average overall rate of sexual protection was 8.1% (s.d. = 19.1) and more than one-third of all sex acts (mean = 37.3%, s.d. = 28.8) entailed internal ejaculation. HIV-positive men used condoms less frequently than HIV-negative men did (5.2% versus 12.2%, *t* = 3.17, *P* = .002). Both groups reported comparable rates of sex involving internal ejaculation (39.6% versus 33.9%, *t* = 1.67, n.s.).

The large majority of the men who had engaged in oral sex reported no condom use during this behavior (89.7%, CI_95_ = 86.2–93.2%). The overall rate of engaging in protected oral sex was 4.1% (s.d. = 16.6). Approximately one-quarter of all oral sex acts resulted in the recipient partner accepting semen directly into the mouth or throat (mean = 27.7%, s.d. = 32.2). All of these figures were comparable for insertive and receptive oral sex, and no significant differences were found based on HIV serostatus.

Most of the men who had engaged in anal sex reported no condom use during this behavior (65.8%, CI_95_ = 60.2–71.5%). Zero condom use during anal sex was more common among HIV-positive men than it was among HIV-negative men (71.4% versus 57.7%, *χ* ^2^_1df_ = 5.54, *P* = .019). The overall rate of condom use during anal sex was 17.2% (s.d. = 31.9). This, too, differed for HIV-positive and HIV-negative men (10.5% versus 26.9%, *t* = 4.31, *P* < .001). More than one-half of all anal sex acts resulted in the recipient partner accepting semen directly into the anus (mean = 52.6%, s.d. = 37.8), with HIV-positive men being more likely than their HIV-negative counterparts to engage in this behavior (57.8% versus 45.3%, *t* = 2.72, *P* = .007). 

As Table 2 shows, double simultaneous penile-anal penetration (i.e., double fucking) was reported during the month prior to interview by approximately one-sixth of the men participating in *The Bareback Project* (15.9%, CI_95_ = 11.8–20.1%). 10.2% of the men reported having engaged in this behavior insertively (CI_95_ = 6.7–13.6%), with internal ejaculation occurring for one or both insertive partners 66.7% of the time (CI_95_ = 49.8–83.5%). 9.5% of the men indicated that they had engaged in double fucking receptively during the month prior to interview (CI_95_ = 6.1–12.8%), and the large majority of these instances (82.1%, CI_95_ = 68.0–96.3%) involved one or both of the insertive partners ejaculating into the recipient man's anus. Analyses comparing HIV-positive and HIV-negative men (see Table 3) revealed that HIV-positive men were almost three times more likely to engage in insertive double fucking than HIV-negative men were (13.7% versus 5.0%, *χ* ^2^_1df_ = 5.92, *P* = .015). HIV-positive men who engaged in receptive double fucking were considerably more likely than their HIV-negative counterparts to engage in this behavior when it entailed internal ejaculation (90.5% versus 57.1%, *χ* ^2^_1df_ = 3.98, *P* = .046).

Oral-anal contact (i.e., rimming) was a common practice among men in this study (82.4%, CI_95_ = 78.0–86.7%), with 66.4% of the men indicating that they had placed their mouth or tongue onto or into a sex partner's anus during the previous month (CI_95_ = 61.0–71.8) and 65.4% reporting that someone had done that to them (CI_95_ = 60.0–70.8%). As Table 3 shows, HIV-positive men were more likely than HIV-negative men to report engaging in receptive oral-anal contact during the previous month (70.9% versus 57.5%, *χ* ^2^_1df_ = 5.65, *P* = .018). Moreover, 16.6% of the sexually active men in the study (or 25.0% of those who acknowledged having been the insertive partner in oral-anal contact) said that they took this one step further by eating semen out of the anus that they were rimming—a practice referred to as felching. Most of these men (63.3% of them or 10.5% of the sexually active sample) reported taking this activity one step further still by then sharing that semen with a sex partner by kissing or drooling the semen into another man's mouth—a practice referred to as snowballing. These practices were fairly comparable among HIV-negative and HIV-positive men alike.

Less commonly reported was sharing of sex toys, such as dildos or butt plugs. Although approximately one-third (33.2%, CI_95_ = 27.8–38.6%) of the sexually active men said that they had used sex toys during the month prior to interview, relatively few of these men (19.4% of them, CI_95_ = 11.6–27.2%, or 6.4% of the men in the study) said that they recently shared a used, uncleaned sex toy with a sex partner. Taking a sex toy from a sex partner who had just used it, and then using it on oneself without cleaning it first, was reported by 3.7% of the men in the study (CI_95_ = 1.6–5.9%) (i.e., 11.2% of those who reported any recent sex toy use). Using a sex toy and then giving it to a sex partner to use without cleaning it first was reported by 5.1% of the respondents (CI_95_ = 2.6–7.6%) (i.e., 15.3% of those who reported any recent sex toy use).

Approximately one-quarter of the sexually active men (23.7%, CI_95_ = 18.9–28.6%) in *The Bareback Project *said that, during the month prior to interview, they had invited several other men to a sexual encounter with the express purpose being to use one of their sex partners or in the alternative, that they had allowed a sex partner to invite other people to a sexual encounter with the express purpose to use them sexually. These behaviors are known as “pimping out” or “being pimped out by” a sex partner, and ordinarily they entail situations in which the inviting partner and the persons he has invited take turns using the “pimped out” partner for their sexual gratification without the “pimped out” partner having much, if any, say in what is done to him sexually. These practices were more common among HIV-positive men than among HIV-negative men (28.0% versus 17.5%, *χ* ^2^_1df_ = 4.34, *P* = .037). Nearly one-fifth of the men in the study (19.3%, CI_95_ = 14.8–23.8%) said that, in the previous month, at least one of their sex partners had done this to them, and about one-half as many (10.5%, CI_95_ = 7.0–14.0%) said that they had done this to someone with whom they had sex.

Nearly one-half of the men participating in the study (47.1%, CI_95_ = 41.4–52.8%) said that, during the month prior to interview, they had engaged in multiple-partner sex (i.e., three or more partners having sex together). The large majority of these men (92.8% of them or 43.7% of the total sample, CI_95_ = 38.1–49.4%) said that this took the form of three-way sexual encounters, and about one-half (56.8% of the men reporting multiple-partner sex or 26.8% of the sexually active men in the study, CI_95_ = 21.7–31.8%) said that this took the form of larger group sexual encounters or orgies. Condom use during multiple-partner sexual encounters tended to be slightly greater than it was overall for other types of sexual behaviors/scenarios, but was still very low: 13.0% of the three-way sexual encounters and 19.1% of the larger-group sexual encounters (accounting for 15.4% of all multiple-partner sexual encounters) involved the use of condoms by one or more of the participants.

These various multiple-partner sex measures all differed greatly based on men's HIV serostatus. Compared to their HIV-negative counterparts, HIV-positive men were more likely to engage in any type of multiple-partner sexual encounter (56.6% versus 33.3%, *χ* ^2^_1df_ = 5.43, *P* < .001), a three-way sexual encounter (52.6% versus 30.8%, *χ* ^2^_1df_ = 13.67, *P* < .001), a three-way sexual encounter involving unprotected sex (49.1% versus 24.2%, *χ* ^2^_1df_ = 18.67, *P* < .001), a larger-group sexual encounter (34.9% versus 15.0%, *χ* ^2^_1df_ = 14.32, *P* < .001), and a larger-group sexual encounter involving unprotected sex (30.3% versus 11.7%, *χ* ^2^_1df_ = 14.06, *P* < .001). Moreover, rates of condom use during these multiple-partner sexual encounters differed for HIV-positive and HIV-negative men. Overall rates of condom use were significantly lower among the former than among the latter (11.3% versus 25.7%, *t* = 2.49, *P* = .014), in large part due to the intergroup differences in protection rates during three-way sexual encounters (8.2% versus 25.0%, *t* = 2.81, *P* = .006).

Approximately one-half (51.2%, CI_95_ = 46.5–56.9%) of the sexually active men said that they had engaged in anonymous sex during the month prior to interview, with this practice being more common among HIV-positive men than it was among HIV-negative men (57.7% versus 41.7%, *χ* ^2^_1df_ = 7.34, *P* = .007). Men who had engaged in recent anonymous sex did so an average of 7.8 times (s.d. = 12.5), and this figure was comparable for HIV-positive and HIV-negative men (7.7 versus 8.0, *t* = 0.16, n.s.).

### 3.4. Drug Use Behaviors

Table 4 summarizes findings for substance use and abuse among men in the sample, and Table 5 provides similar information with comparisons for HIV-negative and HIV-positive men. Most of the men participating in *The Bareback Project *(60.1%, CI_95_ = 54.8–65.4%) reported having used at least one illegal drug during the month prior to interview, and more than one-third of *these *men (38.3% of them or 23.0% of the total sample, CI_95_ = 18.4–27.5%) said that their use entailed the consumption of a drug that was “harder” than marijuana. These figures were similar for HIV-negative and HIV-positive men. The most common drugs of recent use were marijuana (51.1%, CI_95_ = 46.7–56.4%), methamphetamine (16.0%, CI_95_ = 12.1–20.0%), and powdered cocaine (4.5%, CI_95_ = 2.3–6.8%). Comparisons based on HIV serostatus revealed that HIV-positive men were more likely than their HIV-negative counterparts to report recent methamphetamine use (21.4% versus 8.2%, *χ* ^2^_1df_ = 10.48, *P* = .001) and recent use of a club drug other than ecstasy (4.6% versus 0.7%, *χ* ^2^_1df_ = 4.05, *P* = .044). Approximately one man in sixteen (6.0%, CI_95_ = 3.5–8.6%) said that he had injected a drug during the previous 30 days, and this behavior was more common among HIV-positive men than it was among HIV-negative men (10.2% versus 0%).

More than one-quarter of the men taking part in the study (27.8%, CI_95_ = 23.0–32.6%) said that their recent substance use had caused them to experience at least one drug-related problem and/or symptom of drug dependence, and the large majority of *these *men (92.9% of them or 26.0% of the total sample) reported experiencing two or more such problems/symptoms during the preceding month. The most-commonly-reported substance abuse problems and dependency symptoms were trying to make rules to control when or where one's own drug use would be permitted (16.9%, CI_95_ = 12.9–21.0%), continued substance use despite experiencing drug-related depression (9.4%, CI_95_ = 6.2–12.5%), losing interest in friends or activities as a result of drug (ab)use (8.5%, CI_95_ = 5.5–11.5%), needing to use more of a substance in order to get the same high as previously obtained (7.0%, CI_95_ = 4.2–9.7%), and an inability to quit or cut down on one's drug use (6.3%, CI_95_ = 3.8–9.0%).

When asked about their preferences for having sex while high or sober, most study participants (55.4%, CI_95_ = 50.1–60.8%) said that they would prefer to have sex while under the influence of alcohol and/or an illegal drug. This preference was even more pronounced among men who were infected with HIV than among those who were not (64.3% versus 42.7%, *χ* ^2^_1df_ = 15.22, *P* < .001). The large majority of the persons preferring to have sex while “under the influence” (85.9% of them or 47.6% of the total sample, CI_95_ = 42.2–53.0%) said that they would prefer to have sex while under the influence of at least one illegal drug, and most of *these *persons (64.5% of them or 30.7% of the total sample, CI_95_ = 25.8–35.7%) said that they would prefer this drug to be something “harder” than marijuana. Moreover, 41.0% of respondents indicated a preference for both they themselves and their sex partners to be “under the influence” during sex (CI_95_ = 35.7–46.2%). This preference was more common among HIV-infected men than it was among HIV-uninfected men (49.5% versus 28.7%, *χ* ^2^_1df_ = 14.38, *P* < .001). Most of the men who preferred to have sexual relations while both they themselves and their sex partner(s) were “under the influence” (72.8% of them or 29.8% of the sample, CI_95_ = 24.9–34.7%) preferred both they themselves and their sex partners to be high on an illegal drug during sex, with one-half of these individuals (14.5% of the total sample, CI_95_ = 10.7–18.2%) expressing a desire for both partners to be high on a “harder” drug than marijuana. 

To a great extent, men's preferences for having sex while “under the influence” were consistent with their practices. More than one-half of the sexually active study participants (52.4%, CI_95_ = 46.7–58.1%) said that they had engaged in sex while high on alcohol *and/or *an illegal drug during the 30 days prior to interview. This practice was more common among HIV-positive men than it was among HIV-negative men (57.7% versus 44.5%, *χ* ^2^_1df_ = 4.93, *P* = .026). More than one-third of the men in the study (39.1%, CI_95_ = 33.5–44.7%) reported having engaged in sex while high on an illegal drug, and about one-half of *these *men (58.1% of them, CI_95_ = 48.7–67.5%, or 20.7% of the total sample) said that that involved the use of a drug that was “harder” than marijuana.

## 4. Discussion

A number of interesting findings were revealed by this research. First, far from merely using the Internet for pure fantasy or cybersex purposes, during the year prior to interview, the large majority of the sexually active men participating in this study (98.0%, CI_95_ = 96.3–99.6%) actually met in person at least one sex partner originally identified via the Internet (mean = 35.6, SD = 57.6). Moreover, the large majority of the sexually active men participating in *The Bareback Project *reported having had multiple sex partners during the month prior to interview, with the “average” man reporting having had 11 recent sex partners. Other researchers have found a relationship between having multiple sex partners and elevated risk for HIV [24–26]. The present study is consistent with those findings. Compounding this problem, participants in *The Bareback Project *met a substantial proportion of their recent sex partners online, typically after having had relatively brief discussions with them. Most of their sex partners were, therefore, either strangers to them or persons about whom they knew little and with whom they typically engaged in minimal amounts of dialog prior to agreeing to meet for sex. Indeed, qualitative data from the study (not previously discussed) indicated that, in many instances, men in this study did little more than reading someone's profile, checking that person's online photograph(s) to determine their level of sexual interest in that person and then contacting the person with a sexual invitation to “hook up.” Research has shown that partner communication is related inversely to HIV risk involvement [27, 28]. Additional research has shown that sexual risk taking tends to be greater among MSM who meet their sex partners online [1, 7], with this elevated risk oftentimes being attributed, at least in part, to trusting information provided in online profiles and men's tendency not to engage in safer sex discussions with the men they meet online.

Intervention programs targeting risk-seeking MSM such as those who participated in the present study might use several different approaches to trying to reduce risk in this population. For example, they might work with men to reduce their number of sex partners, perhaps suggesting that they develop longer-term “friends with benefits” or “fuck buddies” arrangements with a smaller number of sex partners whom they can get to know better in terms of their sexual health rather than always looking for new partners about whom they know comparatively little with regard to their sexual health. This is purely a harm reduction approach rather than a risk-elimination approach to HIV risk reduction, but the former is likely to be more realistic than the latter. Another strategy might be to work with MSM who meet many of their sex partners online to teach them strategies that they can use to reduce their risk for acquiring or transmitting HIV with partners met this way. For example, interventionists could do more to educate men about the truthfulness (or lack thereof) of men's online profiles. Although many men realize that others are not always truthful about what they say about themselves online, data from *The Bareback Project* indicate that 24.2% of the men believed that online profiles are “very accurate” or “fairly accurate” with regard to the information they contain about a person's HIV serostatus. More education and skills building about how to overcome the occasional lack of truthfulness that pervades many men's online profiles and sexual advertisements might be an effective way of enabling some men to reduce their risk for HIV. This could include intervention components teaching improved partner communication skills.

Also worth considering would be the development and implementation of Internet-based educational and intervention efforts targeting men such as those who participated in *The Bareback Project*. In order to have an honest chance at succeeding, such endeavors would have to be engaging and consist of messages that risk-seeking MSM perceive to be helpful and practical in their lives. Utilizing sexually appealing men as “eye catchers,” particularly on the first web page that men would encounter when visiting these educational/intervention programs' websites, is likely to be an effective technique at garnering their interest and willingness to visit other areas of the website. The community-based HIV prevention group DCFukit (see http://www.dcfukit.org) has utilized this strategy quite effectively in promoting its safer-sex kits and providing MSM with detailed, useful HIV information online. This organization's website is sleek, has a contemporary feeling to it, and is visually appealing (with plenty of photos of shirtless and partly- to mostly-naked men of various ages and races) and has even involved members of the gay pornography industry (in various stages of undress) in providing some of its website's online HIV prevention messages. Any Internet-based HIV prevention/intervention campaign designed to reach risk-seeking MSM should take into account what it takes to capture *and then maintain *the attention of members of this population, as this will be key if the endeavor is to have a chance at being effective. Recent research has shown that including HIV-positive persons' opinions and perspectives on website appeal and utility can be an effective strategy to maximize the potential usefulness of such an approach to providing HIV information [29]. Undoubtedly, this will apply to risk-seeking MSM as well.

Along these same lines, HIV education/intervention projects targeting risk-seeking MSM also might consider availing themselves of emergent technology when providing their messages for MSM. For example, recent evidence has been provided to suggest that men, especially young men, may be amenable to health messages provided via podcasts [30, 31]. As another example, only recently have researchers begun to explore the usefulness of providing health information and prevention content via text messaging, mobile phones, and the so-called smart phones [32]. Early results from these projects are promising particularly for the provision of sexual health and HIV-related information to young adults [33]. How effective these approaches can be at reaching risk-involved MSM remains to be seen, but initial study findings suggest that they are well worth exploring. Research will need to be conducted with regard to whether the use of emergent technologies such as these can be effective at reaching MSM of all ages, all races, and so forth or whether certain subgroups respond better than others to this type of HIV education/intervention approach. This would be a fruitful avenue for future research to pursue.

Another important finding coming from the present study is that a sizable proportion of oral and anal sex acts reported by the men in this study entailed internal ejaculation. The large majority of oral sex acts did not involve the use of a condom, and approximately one-quarter of the unprotected oral sex involved ejaculation into the mouth or throat. Nearly two-thirds of all anal sex acts involved no condom use, and more than one-half of these acts entailed ejaculation directly inside of the anus. Although it is a misconception that sex among MSM almost always involves internal ejaculation, it is nonetheless true that a sizable proportion of these men's sex acts *do *entail the sharing of semen between partners. The main implication of this finding is straightforward: interventionists working with unprotected sex-seeking MSM, such as those who took part in the present study, must find ways to increase condom usage among members of this population or decrease the frequency with which they engage in sexual relations involving internal ejaculation, or both.

Regarding the latter, it is likely that little can be done. Interventionists could work with MSM to encourage them to withdraw the penis (or to have their partners do so) prior to internal ejaculation. This is a practice that has been shown to reduce men's risk for HIV [34]. Another potential approach would be to encourage men to allow their partners to ejaculate *on *them rather than *inside *of them or to ejaculate onto their partners externally rather than internally. Although this would be unpalatable to many of the men who self-identify as “cum lovers” who specifically enjoy the taste or feel of semen (which was true for 60.7% of the men in this study), some might find it to be an acceptable compromise at least some of the time. It is a textbook example of the value of advocating a harm reduction approach, particularly when extinction of a particular behavior (in this instance, the risky practice of having sex involving internal ejaculation) is not a feasible or realistic goal. 

Regarding the former, intervention strategies must consider the question of how to make condoms more appealing and less unpleasant to men who prefer intentional condomless sex. Previous research suggests that highlighting the sexual/sensory aspects of condom use and eroticizing safer sex might help increase condom use among MSM [35]. A number of community-based HIV prevention, education, and intervention programs around the United States have offered workshops about eroticizing safer sex, in an effort to teach members of the MSM community about specific strategies that can be undertaken to make condom use and other safer sex strategies more palatable. Programs such as those offered by Gay Men's Health Crisis in New York City [36], the Howard Brown Health Center in Chicago, and Project AIDS Resources and Knowledge (Project ARK) in St. Louis are to be applauded, as are population-specific approaches such as AIDS Project Los Angeles' Red Circle Project (targeting safer sex among Native Americans) and Bockting et al.'s [37] program targeting safer sex among transgendered persons. Likewise, in recent years, websites dedicated to promoting erotic safer sex have begun to appear on the Internet, and the present author believes that they offer great promise in combating HIV risk taking among MSM. Excellent examples of this may be found in the Washington, DC-based group's DCFukit website, at http://www.dcfukit.org. Finding innovative ways to eroticize safer sex may be an important approach to changing how MSM think about condom use, and that, in turn, is likely to be an effective way of reducing their involvement in risky sexual practices.

Another finding obtained in the present study that is well worth highlighting is that unprotected oral sex and unprotected anal sex were by no means the only HIV risk behaviors in which study participants had been engaging. Men fairly commonly reported recently having engaged in such risky practices as simultaneous double-penile anal penetration (a.k.a. “double fucking,” 15.9%), eating semen out of a man's anus (a.k.a. “felching,” 16.6%), having sex while under the influence of alcohol and/or other drugs (52.2%), “pimping out” a sex partner or “being pimped out” by a sex partner (23.7%), having multiple-partner sex (47.1%), and engaging in anonymous sex (51.2%). Other studies as well have reported these practices to be commonplace among MSM, including published reports focusing on the prevalence of felching (which was remarkably similar to that found in the present study; see [38]), the prevalence of engaging in multiple-partner sex (which was quite similar to that found in the present study; see [39]) and high-risk sex during multiple-partner sexual encounters [40, 41], the prevalence of engaging in anonymous sex (which again was similar to that found in the present study; see [42]), and the common cooccurrence of having sex and using alcohol and/or other drugs [43–45]. In order to be effective in their efforts to combat HIV among MSM such as those who participated in *The Bareback Project*, interventionists will have to develop specific strategies to address the psychosexual needs attendant to each of these particular behaviors. The strategies that prove to be effective at reducing men's involvement in sharing sex toys, for example, are not likely to be equally effective at reducing multiple-partner sexual encounters because the behaviors themselves are very different and the arousal elements that these behaviors satisfy are also very different. Kalichman and Grebler [25] have written about the need to address having multiple concurrent sex partners. Klein [46] has spoken about strategies that might be employed to reduce the risks associated with felching practices among MSM. Fernández and colleagues [47] and Schönnesson and colleagues [48] discussed how interventions might target substance abuse-related risk practices among MSM. These authors' works demonstrate quite clearly how different the HIV intervention needs are likely to be for MSM-targeted endeavors, based on the specific behavior(s) that these interventions wish to affect. Reviewing writings such as these, and considering them in light of the present study's findings regarding the variety of risky practices in which men engaged, makes one thing abundantly clear: reducing HIV risk among MSM such as those who participated in *The Bareback Project* is going to be a complex process.

Another noteworthy finding documented in the present study was the fact that, on many dimensions of HIV risk, HIV-positive and HIV-negative men differed from one another. Although by no means did the two groups differ from one another in terms of their involvement in all of the risk behaviors examined in this paper, in all instances (without exception) in which a difference was found between the two groups, it was always in the direction of HIV-positive men being more likely to engage in the risk practice than their HIV-negative counterparts. To some extent, this is probably due to a false belief on the part of many HIV-infected men that having HIV means that they no longer need to concern themselves as much about their sexual health practices and their drug use behaviors. From their perspective, they already have HIV, so how much worse can it be/get if they engage in high-risk behaviors? Overlooked by adhering to this perspective, of course, is consideration of becoming infected with a new strain of HIV that is resistant to their current medications, becoming infected with a sexually transmitted infection other than HIV, or the enhanced possibility of infecting one's sex partners with HIV by virtue of practicing these high-risk behaviors.

In recent years, numerous public health efforts focusing on HIV-infected MSM have devoted considerable attention to developing effective strategies that can reduce HIV risk behavior involvement in this population. These initiatives have come to be known as “prevention with positives” programs, in which HIV-positive men (and sometimes their sex partners as well, especially if they are involved in ongoing sexual relationships with these partners) are targeted for enhanced, intensive educational, prevention, and/or intervention messages. Important recent research addressing the importance of these programs has included the works published by Wei and colleagues [49], Hatfield and colleagues [50], Crepaz and colleagues [51], Rutledge [52], and Stall and Van Griensven [53] (among many others), with most of these authors having devoted considerable time and attention to discussing strategies for implementing more effective “prevention with positives” programs and the challenges faced by such programs. The findings obtained in the present study support the need for these types of programs in the ongoing effort to curtail the spread of HIV.

Two additional discussion-worthy findings obtained in the present research both pertain to substance use/abuse. The use of illegal drugs was widespread in this research population, and recent use was much more prevalent among study participants (60.1%) than would be expected among men in the US general population (9.9%) [54]. Moreover, most of the study participants (56.4%) said that they prefer to have sex while under the influence of alcohol and/or illegal drugs rather than while sober, with the large majority of these men (85.9% of them) indicating a preference for having sex while high on an illegal drug. Indeed, more than one-half of the men who took part in this study (52.2%) reported recently having engaged in sexual relations at a time when they were not sober. These findings are of great concern, because there is a well-established association between substance use/abuse and involvement in risky sex [55–57]. Clearly, there is a need for substance abuse prevention education, drug abuse intervention services, and substance abuse treatment among men who use the Internet to find partners for unprotected sex. Other scholars as well have spoken of the need for these types of services among MSM [58–60]; the present study supports their contention. Completing drug treatment has been shown to be effective at helping to reduce HIV risk practices in a variety of population groups [61, 62], including MSM [63, 64]. The key to implementing effective drug abuse prevention and treatment services will be making sure that these programs are sensitive to the needs of gay and bisexual men and that they are designed in a culturally appropriate manner. In recent years, several such programs have evolved around the United States, offering treatment services specifically for gay men due to their unique needs for substance abuse recovery. Examples include Freedom Rings (Jacksonville, FL,), Michael's House (Palm Springs, CA, MSA), Out Interventions (Venice, CA, MSA), Pride Institute (Dallas, TX), and Rainbow Recovery (Laguna Beach, CA, MSA).

### 4.1. Potential Limitations

Before concluding, the author wishes to acknowledge two potential limitations of this research. First, the data in this study are based on uncorroborated self-reports. Therefore, it is unknown whether participants underreported or overreported their involvement in risky behaviors. The self-reported data probably can be trusted, however, as noted by other authors of previous studies with similar populations [65]. This is particularly relevant for self-reported measures that involve relatively small occurrences (e.g., number of times having a particular kind of sex during the previous 30 days), which characterize the substantial majority of the data collected in this study [66]. Other researchers have also commented favorably on the reliability of self-reported information in their studies regarding topics such as condom use [67]. 

A second potential limitation is the possibility of recall bias. For most of the measures used, respondents were asked about their beliefs, attitudes, and behaviors during the past 7 or 30 days. These time frames were chosen specifically: (1) to incorporate a large enough time frame in order to facilitate meaningful variability from person to person, and (2) to minimize recall bias. Although the authors cannot determine the exact extent to which recall bias affected the data, other researchers who have used similar measures have reported that recall bias is sufficiently minimal that its impact upon study findings is likely to be negligible [68, 69]. This seems to be especially true when the recall period is small [70, 71], as was the case for most of the main measures used in the present study. 

A third limitation of this research is the unknown extent to which findings may be generalized. As with many published studies focusing on risk practices among MSM, the present study had a larger-than-expected proportion of well-educated men. HIV-positive men were overrepresented as well, which is not surprising when one considers that the study population was comprised by men seeking unprotected sex online. Although study participants were selected at random from the various websites used for recruitment purposes, there is no way to know how well these individuals represent unprotected sex-seeking MSM more broadly. What the present study population does represent, however, is a sample of risk-seeking MSM who actively use the Internet to identify potential sex partners.

## 5. Conclusion

In conclusion, all of the preceding findings—men's large numbers of recent sex partners, their propensity for initially identifying sex partners online and then meeting them for sex after minimal interaction/conversation, their widespread lack of condom use coupled with a tendency for many men to prefer having sex involving internal ejaculation, the multitude of risky sexual behaviors in which men were engaging, and widespread substance use/abuse in conjunction with sexual encounters—add up to a situation in which men who use the Internet to find partners for unprotected sex are placing themselves and their sex partners at great risk for contracting and/or transmitting HIV. The sheer variety of risky behaviors in which these men engage combine to form a very complicated web of behavioral risks. Each of these risk behaviors fulfills a specific need or set of needs physically, emotionally, and psychosexually, and as a result, each requires a unique approach to intervention and risk reduction. If HIV risk reduction efforts are to be successful among men who use the Internet to locate partners for unprotected sex, they will need to be comprehensive and creative, and capable of disentangling the multitude of risk factors and risk practices that commonly cooccur among men in this population. This will not be an easy task.

## Figures and Tables

**Table 1:** Sample Characteristics.

Characteristic	*N*	%
Age		
18–29	44	13.3
30–39	69	20.8
40–49	109	32.9
50–59	81	24.5
60+	28	8.5
Race/ethnicity		
Caucasian	246	74.1
African American	30	9.0
Latino	30	9.0
Asian/Pacific Islander	8	2.4
Native American/Native Alaskan	1	0.3
Biracial/multiracial	17	5.1
Educational attainment		
High school graduate or less	48	14.5
Some college	114	34.3
College graduate	96	28.9
Postgraduate	74	22.3
Population density in area of residence		
Rural (<250 persons per square mile)	76	22.9
Urban (1,000+ persons per square mile)	198	59.6
Low density (1,000 to 2,500 persons)	(53)	(16.0)
Medium density (2,501 persons to 5,000 persons)	(67)	(33.8)
High density (5,001+ persons)	(88)	(44.4)
Relationship status		
Married or “involved”	87	26.2
Single	245	73.8
HIV serostatus		
Negative	128	38.6
Positive	196	59.0
Do not know	8	2.4
Sexual orientation		
Gay	297	89.5
Bisexual	34	10.2
Sexual role identity		
Total top	54	16.3
Versatile top	62	18.7
Versatile	60	18.1
Versatile bottom	92	27.7
Total bottom	64	19.3
Internet use—looking specifically for bareback partners		
Once or twice a week	105	31.7
<30 minutes each day	(74)	(70.5)
30 to 60 minutes each day	(24)	(22.9)
61 to 120 minutes each day	(5)	(4.8)
121+ minutes each day	(2)	(1.9)
Three to six times a week	121	36.6
<30 minutes each day	(74)	(61.2)
30 to 60 minutes each day	(33)	(27.3)
61 to 120 minutes each day	(8)	(6.6)
121+ minutes each day	(6)	(5.0)
Daily	105	31.7
<30 minutes each day	(47)	(44.8)
30 to 60 minutes each day	(29)	(27.6)
61 to 120 minutes each day	(12)	(11.4)
121+ minutes each day	(17)	(16.2)
Internet use—looking for sex partners, not bareback specific (in addition to looking for bareback partners)		
Once or twice a week	53	16.0
<30 minutes each day	(43)	(81.1)
30 to 60 minutes each day	(7)	(13.2)
61 to 120 minutes each day	(1)	(1.9)
121+ minutes each day	(2)	(3.8)
Three to six times a week	127	38.4
<30 minutes each day	(29)	(22.8)
30 to 60 minutes each day	(63)	(49.6)
61 to 120 minutes each day	(26)	(20.5)
121+ minutes each day	(9)	(7.1)
Daily	151	45.6
<30 minutes each day	(33)	(21.9)
30 to 60 minutes each day	(47)	(31.1)
61 to 120 minutes each day	(29)	(19.2)
121+ minutes each day	(41)	(27.3)

**Table 2:** Prevalence of selected sexual behaviors among sexually active men.

Sexual behavior	*N*	%
Oral sex—insertive		
No	17	5.8
Yes	277	94.2
With partners met online?		
No	53	19.1
Yes	224	80.9
Any of it unprotected?		
No	23	8.3
Yes	254	91.7
Any of it entailing internal ejaculation?		
No	125	45.1
Yes	152	54.9
Oral sex—receptive		
No	28	9.5
Yes	267	90.5
With partners met online?		
No	59	22.1
Yes	208	77.9
Any of it unprotected?		
No	16	6.0
Yes	251	94.0
Any of it entailing internal ejaculation?		
No	147	55.3
Yes	119	44.7
Anal sex—insertive		
No	106	35.9
Yes	189	64.1
With partners met online?		
No	32	16.9
Yes	157	83.1
Any of it unprotected?		
No	51	27.0
Yes	138	73.0
Any of it entailing internal ejaculation?		
No	58	30.7
Yes	131	69.3
Anal sex—receptive		
No	96	32.5
Yes	199	67.5
With partners met online?		
No	42	21.1
Yes	157	78.9
Any of it unprotected?		
No	64	32.3
Yes	134	67.7
Any of it entailing internal ejaculation?		
No	51	25.6
Yes	148	74.4
Sex partners in past 30 days		
1	38	12.9
2–5	109	36.9
6–10	52	17.6
11–20	61	20.7
21–30	16	5.4
31+	19	6.4
Double fucking—insertive		
No	265	89.8
Yes	30	10.2
Any of it unprotected?		
No	4	13.3
Yes	26	86.7
Any of it entailing internal ejaculation?		
No	10	33.3
Yes	20	66.7
Double fucking—receptive		
No	267	90.2
Yes	29	9.8
Any of it unprotected?		
No	5	17.2
Yes	24	82.8
Any of it entailing internal ejaculation?		
No	5	17.2
Yes	24	82.8
Oral-anal contact—insertive		
No	99	33.6
Yes	196	66.4
Any of it involving felching?		
No	147	75.0
Yes	49	25.0
Any of it involving snowballing?		
No	18	36.7
Yes	31	63.3
Oral-anal contact—receptive		
No	102	34.6
Yes	193	65.4
Sharing of used, uncleaned sex toys		
No	79	80.6
Yes	19	19.4
Any pimping out or being pimped out sexually?		
No	225	76.3
Yes	70	23.7
Multiple partner sexual encounters		
No	156	52.9
Yes	139	47.1
Any of it involving three ways?		
No	10	7.8
Yes	129	92.1
Any of it involving unprotected sex?		
No	24	18.6
Yes	105	81.4
Any of it involving larger-group encounters?		
No	60	43.2
Yes	79	56.8
Any of it involving unprotected sex?		
No	20	25.3
Yes	59	74.7
Anonymous sex encounters		
No	144	48.8
Yes	151	51.2

**Table 3:** Comparison of HIV-negative and HIV-positive men's involvement in selected sexual behaviors among sexually active men.

Sexual behavior	HIV-negative (% yes)	HIV-positive (% yes)	*P* = | *x*|
Oral sex—insertive			
Past 30 days	92.5	95.4	n.s.
With partners met online?	80.2	80.8	n.s.
Any of it unprotected?	90.8	94.9	n.s.
Any of it entailing internal ejaculation?	46.9	59.9	.033
Oral sex—receptive			
Past 30 days	94.2	88.0	n.s.
With partners met online?	78.8	77.3	n.s.
Any of it unprotected?	91.7	86.9	n.s.
Any of it entailing internal ejaculation?	43.8	45.5	n.s.
Anal sex—insertive			
Past 30 days	60.8	66.3	n.s.
With partners met online?	83.6	82.8	n.s.
Any of it unprotected?	50.8	64.0	.024
Any of it entailing internal ejaculation?	63.0	73.3	n.s.
Anal sex—receptive			
Past 30 days	60.8	72.0	.044
With partners met online?	80.8	77.8	n.s.
Any of it unprotected?	51.7	69.1	.002
Any of it entailing internal ejaculation?	65.8	79.4	.034
Sex partners in past 30 days			.002
1	14.2	12.0	
2–5	49.2	28.6	
6–10	15.0	19.4	
11–20	14.2	25.1	
21+	7.5	14.9	
Double Fucking—Insertive			
Past 30 days	5.0	13.7	.015
Any of it unprotected?	98.3	98.9	n.s.
Any of it entailing internal ejaculation?	66.7	66.7	n.s.
Double fucking—receptive			
Past 30 days	5.8	12.0	n.s.
Any of it unprotected?	97.5	99.4	n.s.
Any of it entailing internal ejaculation?	57.1	90.5	.046
Oral-anal contact—insertive			
Past 30 days	60.8	70.3	n.s.
Any of it involving felching?	24.7	25.2	n.s.
Any felching involving snowballing?	55.6	67.7	n.s.
Oral-anal contact—receptive	57.5	70.9	.018
Sharing of used, uncleaned sex toys	22.6	17.9	n.s.
Pimping out or being pimped out sexually	17.5	28.0	.037
Multiple-partner sexual encounters			
Past 30 days	33.3	56.6	<.001
Any of it involving three ways?	30.8	52.6	<.001
Any three ways involving unprotected sex?	24.2	49.1	<.001
Any of it involving larger-group encounters?	15.0	34.9	<.001
Any larger group encounters involving unprotected sex?	11.7	30.3	<.001
Anonymous sex encounters	41.7	57.7	.007

**Table 4:** Substance use behaviors.

Substance use behavior	*N*	%
Any illegal drug use in past 30 days?		
No	132	39.9
Yes	199	60.1
Which drug(s)?		
Marijuana	108	54.3
Cocaine	15	7.5
Crack	8	4.0
Heroin or other opiates	3	1.5
Hallucinogens	0	0.0
Ecstasy	8	4.0
Club drugs other than ecstasy	10	5.0
Methamphetamine	53	26.6
Sedatives or depressants	11	5.5
Any drug-related problems in past 30 days?		
No	239	72.2
Yes	92	27.8
How many? (out of 13 listed)		
1	40	43.5
2	22	23.9
3	11	12.0
4 or more	19	20.7
Any sex while “under the influence” in past 30 days?		
No	175	52.9
Yes	156	47.1
Which drug(s)? (top 3 listed; all others less common)		
Alcohol	89	57.1
Marijuana	78	50.0
Methamphetamine	51	32.7
Prefer to have sex while “under the influence”?		
No	148	44.6
Yes	184	55.4
Which drug(s)? (top 4 listed; all others less common)		
Alcohol	60	32.6
Marijuana	96	52.2
Ecstasy	37	20.1
Methamphetamine	60	32.6
Ever been in drug treatment?		
No	271	81.6
Yes	60	18.4
More than once?		
No	35	58.3
Yes	25	41.7

**Table 5:** Differences between HIV-positive and HIV-negative men in substance use behaviors.

Substance use behavior	HIV-negative (% yes)	HIV-positive (% yes)	*P* = | *x*|
Any illegal drug use in past 30 days?	57.8	61.7	n.s.
Among recent users of any illegal drugs, which drug(s)?			
Marijuana	52.6	50.0	n.s.
Cocaine	3.0	5.6	n.s.
Crack	1.5	3.1	n.s.
Heroin or other opiates	0.7	1.0	n.s.
Hallucinogens	0.0	0.0	n.s.
Ecstasy	1.5	3.1	n.s.
Club drugs other than ecstasy	0.7	4.6	.044
Methamphetamine	8.2	21.4	.001
Sedatives or depressants	3.0	3.6	n.s.
Any drug problems in past 30 days?	25.9	29.1	n.s.
Among people with any recent drug problems, how many? (out of 13 listed)			n.s.
1	45.7	42.1	
2	22.9	24.6	
3	11.4	12.3	
4 or more	20.0	21.1	
Any sex while “under the influence” in past 30 days?	44.5	57.7	.026
Among people with any recent sex while “under the influence,” which drug(s)? (top 3 listed; all others less common)			
Alcohol	29.1	31.4	n.s.
Marijuana	17.7	32.6	.004
Methamphetamine	25.7	43.8	n.s.
Prefer to have sex while “under the influence”?	42.7	64.3	<.001
Among those preferring to have sex while “under the influence,” which drug(s)? (top 4 listed; all others less common)			
Alcohol	17.7	17.4	n.s.
Marijuana	19.9	35.2	.002
Ecstasy	11.0	11.2	n.s.
Methamphetamine	9.6	24.0	<.001
Ever been in drug treatment?	15.6	19.9	n.s.
Among those who have been in treatment before, been in treatment more than once?	57.1	33.3	n.s.

## Endnotes

1. Due to differences from website to website in terms of the information made available to users about whether or not their emails were received and read, or whether they were removed by the web host's system prior to being read by the intended recipient, it is impossible to compute an accurate participation rate for *The Bareback Project*. Based on websites where enough information was available to users to allow for the participation rate to be calculated, the response rate was slightly greater than 10%. This could raise concern of selection bias and, therefore, the representativeness of the sample. Although it is difficult to be certain that the men who participated represent the men who did not, there is compelling evidence to suggest that differences between the two groups are minimal. Before *The Bareback Project* was started, the principal investigator conducted a large-scale content analysis with a random national sample of one of the main websites used by men to meet other men seeking unprotected sex partners (for additional information, see [19, 38, 72, 73]). The demographic composition of that sample and the one obtained in *The Bareback Project *closely match one another in terms of age representation, racial group composition, sexual orientation, and rural/suburban/urban location of residence. The two samples also resemble one another closely in terms of the types of sexual practices that men sought. The similarity of the two samples suggests that men who chose to participate in the present study represent those who did not, in terms of identifiable characteristics that are likely to be the best indicators of selection bias. Also, the demographic composition of men in *The Bareback Project* and the demographic composition of the male adult population-at-large are a fair approximation of one another in terms of their age breakdown [74] and rural/suburban/urban location of residence [75]. The present sample is better educated than men in the general population [74] and more likely to be HIV-positive (which is to be expected when one considers the population targeted in the present research).

2. It is interesting to note that the man reporting the largest number of sex partners over the course of his lifetime (*n* = 20, 000) had actually maintained a written diary, which he has since computerized, detailing all of his lifetime sex partners. Ever since the time of his first sexual experience, he had kept detailed records of each different person with whom he had had any type of sexual contact. Therefore, rather than being an overestimation as one might be inclined to expect, the number of partners he reported was very close to accurate. During the interview, he admitted to a slight rounding down of his lifetime number of partners, in the “off chance” that he may have double-counted a few persons accidentally. Also worth noting is the fact that this particular study participant was not the only person interviewed who had maintained sexual records. He was, as it turns out, one of several men who, during their interviews with the research team, casually mentioned keeping such records.
